# A Novel Compound from the Phenylsulfonylpiperazine Class: Evaluation of In Vitro Activity on Luminal Breast Cancer Cells

**DOI:** 10.3390/molecules29184471

**Published:** 2024-09-20

**Authors:** Fernanda Cardoso da Silva, Ana Clara Cassiano Martinho, Helen Soares Valença Ferreira, Raoni Pais Siqueira, Vinicius Marques Arruda, Joyce Ferreira da Costa Guerra, Maria Laura dos Reis de Souza, Emanuelly Silva Landin, Celso de Oliveira Rezende Júnior, Thaise Gonçalves de Araújo

**Affiliations:** 1 Laboratory of Genetics and Biotechnology, Institute of Biotechnology, Universidade Federal de Uberlândia, Patos de Minas 38700-002, MG, Brazil; fernanda.cardoso95@yahoo.com (F.C.d.S.); helensvalenca@gmail.com (H.S.V.F.); raoni.siqueira@ufu.br (R.P.S.); viniciusmarquesarruda@gmail.com (V.M.A.); 2 Laboratory of Drug Candidate Synthesis, Institute of Chemistry, Universidade Federal de Uberlândia, Uberlândia 38400-902, MG, Brazilmarialaura1200@hotmail.com (M.L.d.R.d.S.); emanuelly.landin@ufu.br (E.S.L.); celso@ufu.br (C.d.O.R.J.); 3 Laboratory of Biochemistry, Institute of Biotechnology, Universidade Federal de Uberlândia, Patos de Minas 38700-002, MG, Brazil; joyceguerra@ufu.br; 4 Laboratory of Nanobiotechnology Prof. Dr. Luiz Ricardo Goulart Filho, Institute of Biotechnology, Universidade Federal de Uberlândia, Uberlândia 38405-302, MG, Brazil

**Keywords:** breast cancer, chemotherapy, phenylsulfonylpiperazine, sulfonamides, cytotoxicity

## Abstract

Breast cancer (BC) is the most common cancer in women, and is characterized by its histological and molecular heterogeneity. Luminal BC is an estrogen receptor-positive subtype, with varied clinical courses. Although BC patients are eligible for hormone therapy, both early and late relapses still occur, and thus there is a demand for new cytotoxic and selective treatment strategies for these patients. In the present study, inspired by the structure of phenylsulfonylpiperazine, a series of 20 derivatives were tested in bioassays against MCF7, MDA-MB-231 and MDA-MB-453 BC cells to discover new hit compounds. After 48 h of treatment, 12 derivatives impaired cell viability and presented significant IC_50_ values against at least one of the tumor lineages. Overall, the luminal BC cell line MCF7 was more sensitive to treatments. Compound **3**, (4-(1H-tetrazol-1-yl)phenyl)(4-((4-chlorophenyl)sulfonyl)piperazin-1-yl)methanone, was the most promising, with IC_50_ = 4.48 μM and selective index (SI) = 35.6 in MCF7 cells. Compound **3** also presented significant antimigratory and antiproliferative activities against luminal BC cells, possibly by affecting the expression of genes involved in the epithelial–mesenchymal transition mechanism, upregulating E-Cadherin transcripts (*CDH1*). Our findings suggest that phenylsulfonylpiperazine derivatives are potential candidates for the development of new therapies, especially those targeting luminal BC.

## 1. Introduction

Breast cancer (BC) is the most commonly diagnosed cancer in women worldwide. About 2,308,897 cases were reported, including 665,684 deaths, in 2022 [1]. The survival rates related to this disease have improved over the past few decades, and this improvement has been associated with optimized early diagnosis strategies and less invasive and aggressive therapies [2]. However, the numbers remain alarming, with around 1 million deaths projected by 2040. The reality is even more serious in countries classified with a low or medium Human Development Index (HDI), and global efforts are urgently needed to counteract the growing BC burden [3].

Breast tumors are highly heterogeneous, and in addition to the classic histopathological parameters, tumor size, grade and nodal involvement, molecular subtypes and predictive signatures have proven to be essential for predicting therapeutic response and patient prognosis [4]. Currently, five molecular subtypes are clinically adopted, based on an expression profile consisting of the estrogen receptor (ER), progesterone receptor (PR) and human epidermal growth factor receptor 2 (HER2). Tumors expressing ER and/or PR are categorized as luminal BC, which may or may not express HER2, defined as luminal A-like (strongly ER+ and PR+; HER2−), luminal B-like/HER2− (with ER and PR levels lower than that of luminal A, but a high proliferation index) and luminal B-like/HER2+ (ER and PR levels lower than luminal A; HER+). When the tumors only express HER2, they are deemed to be HER2-enriched; those that do not express ER, PR or HER2 are labelled as triple negative-BC (TNBC) [5]. 

Luminal HER2− BC is the most prevalent subtype, representing around 70% of cases, and thus contributing to the highest number of deaths related to the disease [6]. Systemic adjuvant treatment regimens are based on the risk of disease recurrence [7,8]. Luminal BC has challenged clinical decisions, since it was initially considered to have a good prognosis but has shown a high relapse rate. However, these tumors bear a risk of recurrence that can persist even after 20 years of treatment [9] and it is already known that luminal tumors can cause metastases, accounting for approximately two-thirds of all cases [10], including those with HER2+ molecular characteristics [11]. In this context, new strategies are necessary, especially those that target mechanisms related to the metastatic process and those that inhibit the progression of the disease.

A class of compounds that has been attracting attention includes benzenesulfonamide derivatives, novel synthetic substances derived from sulfonamides [12,13,14] that present distinct biological properties, such as anti-inflammatory [15], antibiotic [16], antioxidant [17], immunosuppressive [18], antiparasitic [19,20] and antitumor properties [14,21,22,23,24,25]. It is noteworthy that sulfonamides have antiproliferative, anti-metastatic, antimigratory and pro-apoptotic activities in tumor cells. The main known mechanisms of action include modulation of the PI3K/AKT/mTOR signaling pathway, inhibition of carbonic anhydrase, regulation of monocarboxylate transporters, interference with microtubule polymerization, an influence on hypoxia-inducible factor 1, interaction with proteins involved in apoptosis, suppression of tumor multidrug resistance and regulation of reactive oxygen species [12,26,27,28,29,30]. Regarding the pressing demand for novel treatments for luminal BC, the aim of this study was to investigate the effects of phenylsulfonylpiperazine derivatives in breast tumors, particularly in luminal BC cells. This study meets an urgent demand regarding luminal tumors that, despite being molecularly favorable, have a clinical outcome that is still uncertain.

## 2. Results

### 2.1. Synthesis

**Scheme 1:**
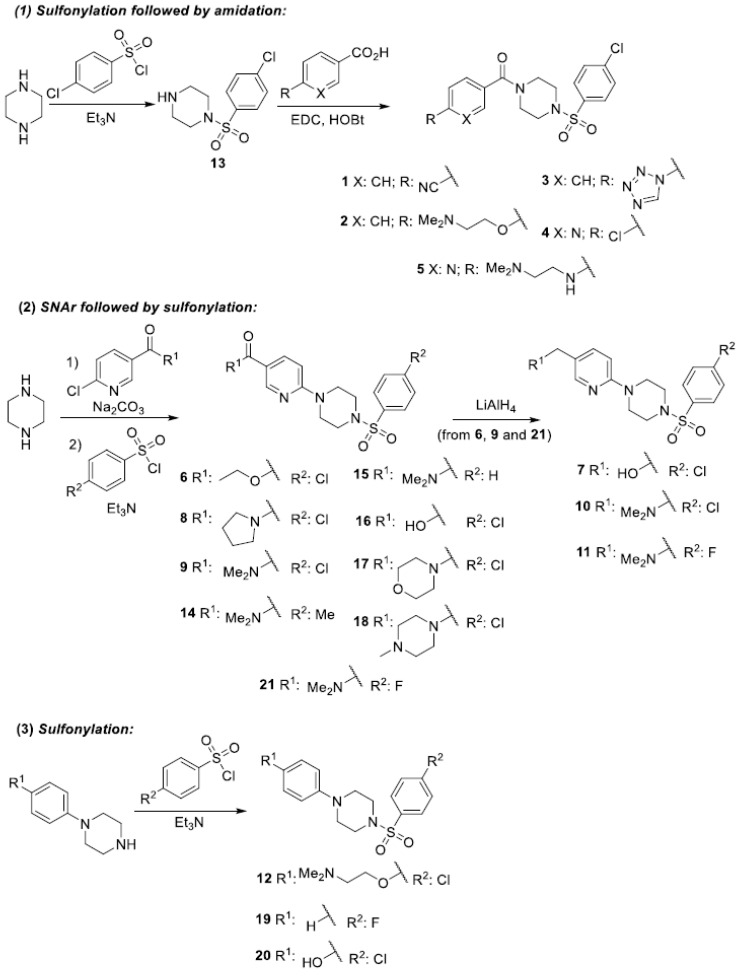
General strategy for the synthesis of compounds **1**–**20**.

The 20 phenylsulfonylpiperazine derivatives were synthesized using three primary synthetic strategies, as shown in Scheme 1. Compounds **1**–**5** and **13** were prepared through a route involving sulfonylation followed by amidation. Compounds **6**–**11** and **14**–**18** were synthesized via nucleophilic aromatic substitution (SNAr) followed by sulfonylation, whereas compounds **12**, **19** and **20** were obtained through a sulfonylation reaction. All compounds were previously characterized using ^1^H and ^13^C nuclear magnetic resonance (NMR) spectroscopy and mass spectrometry [19,20].

### 2.2. Screening for Active Compounds

The cytotoxicity of the phenylsulfonylpiperazine derivatives to mammary cell lines was evaluated against non-tumor cell line MCF-10A and tumor cells MCF7, MDA-MB-453 and MDA-MB-231. A screening of 20 phenylsulfonylpiperazine derivatives revealed that 12 compounds were cytotoxic up to the highest concentration tested (160 μM), as detailed in Table 1 and Supplementary Figure S1. In contrast, eight compounds showed no cytotoxicity, as presented in Table S1 of the Supplementary Materials and Supplementary Figure S2. Compounds **1**, **4**, **5** and **6** were not cytotoxic to the MCF-10A cell line, but in tumor cells, they presented IC_50_ > 50 μM. These findings limit their potential for future pharmacological applications, since high values of IC_50_ highlight the need for high doses of the compound to achieve the desired antitumor effect, which increases the toxicity of the treatment [31]. Compounds **3** and **11** demonstrated the highest cytotoxicity against the MCF7 tumor cell line, with IC_50_ = 4.48 μM and IC_50_ = 20.00 μM, respectively. The SI values were above 35 for **3** and above 8 for **11**. Therefore, compound **3** (Supplementary Figure S3) was chosen for subsequent assays.

### 2.3. Antioxidant Potential

**Figure 1:**
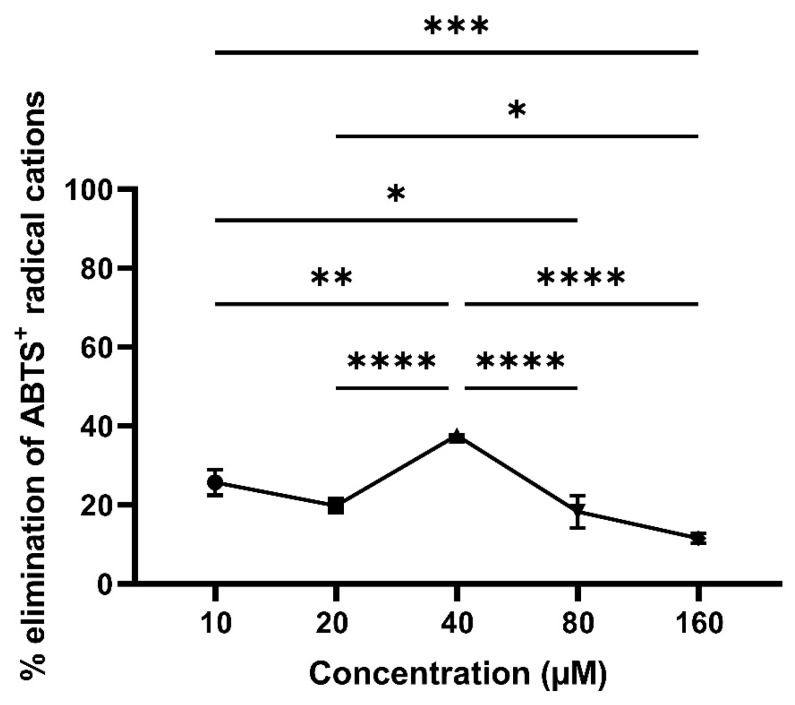
Graphical representation of the antioxidant potential of compound **3**. The ABTS method was performed and given as a percentage of inhibition according to the concentration of the compound. *p* values are represented by * (*p* < 0.05); ** (*p* < 0.01); *** (*p* < 0.001) and **** (*p* < 0.0001) using one-way ANOVA and Tukey’s multiple comparisons test.

The antioxidant activity assay using the ABTS method evaluated whether the cytotoxic action observed for compound **3** is correlated with its oxidizing or antioxidant action. Among the concentrations tested (10, 20, 40, 80 and 160 µM), 40 µM was the one that showed the greatest antioxidant activity (40% inhibition of free radicals), yet it lacked a dose-dependent profile (Figure 1).

### 2.4. Clonogenicity and Migration

**Figure 2:**
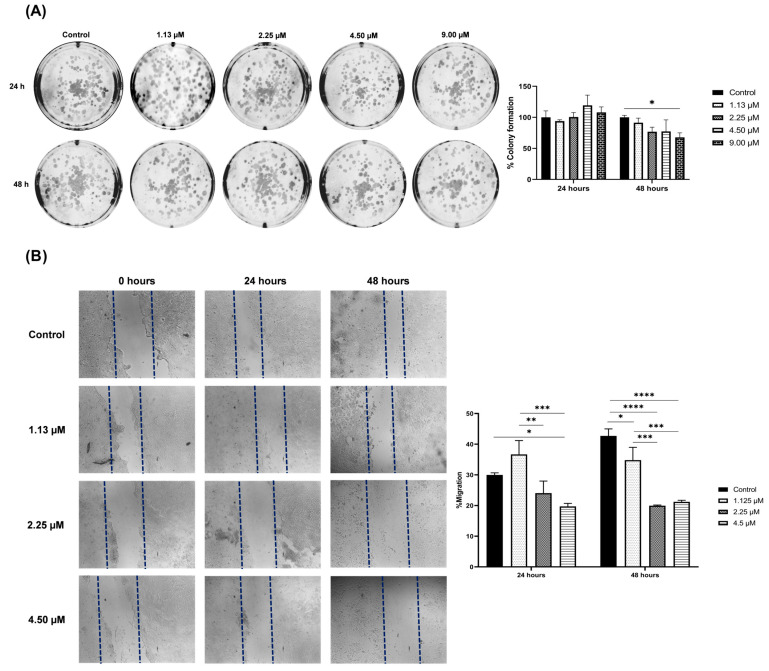
Inhibition of clonogenicity and migration of MCF7 cells treated with different concentrations of compound **3** for 24 and 48 h. (**A**) Colony formation was inhibited only in the highest concentration of compound **3**. (**B**) Cell migration, represented as a percentage, was significantly affected at both treatment times, especially after 48 h. *p* values represented by * (*p* < 0.05); ** (*p* < 0.01); *** (*p* < 0.001) and **** (*p* < 0.0001) using one-way ANOVA and Tukey´s multiple comparisons test.

The clonogenic assay was conducted on the MCF7 cell line to evaluate how compound **3** affects the ability of individual cells to form colonies under in vitro culture conditions. This technique is commonly used to detect and quantify self-renewing mammalian cells in vitro, thus determining the ability of cells to establish themselves in new sites [32]. Cells were treated with compound **3** at concentrations of 1.13, 2.25, 4.50 and 9.0 µM. It was observed that only the treatment with 9.00 µM of compound **3** for 48 h significantly reduced colony formation (Figure 2A). The migration assay, in turn, monitored the recolonization process of the wound area to quantify cell migration after treatment with the compound of interest [33], which is a hallmark during morphogenesis, inflammation and metastasis [34]. Compound **3** significantly inhibited the migration of MCF7 cells when used at concentrations of 2.25 and 4.50 µM for 24 h and at concentrations of 1.13, 2.25 and 4.50 µM after 48 h of treatment (Figure 2B).

### 2.5. Transcriptional Modulation

qPCR assays were then conducted to elucidate the molecular mechanisms modulated by compound **3** in luminal BC cells, MCF7. The expression of the *CDH1*, *ENTPD1*, *ENTPD5*, *KRT10*, *ENTPD2* and *ENTPD3* genes was evaluated. The treatment significantly increased *CDH1* expression by approximately four-fold, as shown in Table 2. 

## 3. Discussion

Chemotherapy has played a central role in the treatment of BC. However, given its heterogeneity and the increase in cases of resistance, there is now a pressing need to synthesize and validate new compounds with antitumor activity [35]. Compounds belonging to the classes of sulfonamides and piperazines have stood out due to their different mechanisms of action, targeting chemoresistant and hormone-resistant cells, expanding their application potential [36,37].

The chemical structure determines the biological activity of chemical compounds [38] and, in our study, the nature and position of the electron-withdrawing and electron-donating functional groups in the nucleus of the compounds impacted the electronic and steric properties, and may have contributed to the observed effects. The initial screening of the 20 compounds by the MTT assay in the four breast cell lines allowed an overview of their cytotoxic action. The literature highlights the importance of evaluating not only their potency, but also their selectivity, especially in complex and heterogeneous diseases such as cancer [39].

Compounds **1**–**5** are derivatives of *p*-chlorobenzenesulfonylpiperazine, featuring an acyl group attached to the piperazine nitrogen. We observed that these compounds were generally non-cytotoxic against the non-tumoral MCF-10A cell line, except for compound **2**, with IC_50_ = 88.20 μM. For tumor cells, compounds **3**, **10** and **12** presented IC_50_ < 50 µM. However, compound **3** exhibited notable cytotoxicity against the MCF7 cell line, with IC_50_ = 4.48 μM and SI > 35.6. Compounds **6**–**11** contain a pyridyl group attached to the piperazine nitrogen. The MCF-10A lineage was more sensitive to this group, with compounds **7**–**9** being more cytotoxic. Compound **11**, in turn, exhibited significant cytotoxicity against the MCF7 tumor cell line, with IC_50_ = 20.00 μM and SI > 8. The sole structural difference between compounds **11** and **10** is the replacement of fluorine to chlorine, which resulted in a three-fold increase in cytotoxicity against MCF7. Compound **9** differs from compound **10** by substituting a carbonyl group to a methylene group. This structural modification led to higher cytotoxicity against non-tumoral MCF-10A cells, and lower cytotoxicity against tumor cells for the carbonylated derivative. Finally, compound **12**, an *N*-phenylpiperazine derivative, was not selective.

Compound **3** emerged as the most promising in the series, displaying low micromolar potency against the MCF7 cell line and a significant SI. The primary structural difference in this compound, compared to the other derivatives, is the presence of a tetrazole ring in the amino fragment. While the other compounds feature sp^3^, sp^2^ and sp nitrogen species in the amino fragment, none of them have the aromatic properties of tetrazole, which is a cyclic, planar and conjugated substituent with three pairs of pi electrons. Furthermore, tetrazole has four nitrogen atoms, which may facilitate intermolecular interactions with a biological target, thereby enhancing the cytotoxicity of compound **3** to the MCF7 cell line. Therefore, compound **3** was chosen for additional assays against the MCF7 lineage, a representative of the BC luminal phenotype.

Luminal BC is a tumor that is associated with a better prognosis and is eligible for hormone therapy. However, the number of cases of metastasis and recurrence is increasing, causing researchers to question the currently adopted compounds and strategies [35,40,41]. The cytotoxic action of compounds from the benzenesulfonamide class has already shown promising results in tumor cells. Gurdal and collaborators [42] evaluated the effects of 19 compounds from the piperazine class, which were also active in the MCF7 lineage. According to the authors, the cytotoxicity of sulfonamides increases when the central structure has a 4-chloro substitution in the benzhydryl moiety. Furthermore, Patel and collaborators pointed out that compounds from the sufonylpiperazine class that have halogenation present greater antitumor potential, while those with a methoxy functional group have protective effects [43]. Sun and collaborators also previously evaluated four compounds obtained from a sulfonamide library in MCF7 cells [44]. However, they were less selective than our compound **3**, which is characterized not only by halogenation and insertion of the -Cl group, but also by the insertion of a tetrazole group, which may have favored intracellular transport.

Subsequently, the antioxidant potential of compound **3** was evaluated. Compounds from the piperazine class have already shown remarkable antioxidant power in eliminating DPPH(·) and ABTS(·+), as they are able to eliminate free radicals [38]. However, the compound evaluated here showed a maximum ABTS inhibition of 40% at a concentration of 40 µM, which suggests that compound **3** exhibits cytotoxic action mediated by a mechanism other than the scavenging of free radicals. For this reason, we analyzed other mechanisms and observed that compound **3** significantly inhibits colony formation and also interferes with the migration of the MCF7 lineage. As for the mechanisms involved, the compound increased *CDH1* transcriptional levels. E-cadherin is an essential component of the cytoskeleton acting on adherens junctions [45]. The interaction between cells plays a crucial role in cell adhesion and the regulation of proliferation, especially when cells reach confluence [46]. Furthermore, loss of E-cadherin expression is a poor prognostic factor in BC and is downregulated in tamoxifen-resistant cells [47,48]. Therefore, by increasing *CDH1* transcripts, compound **3** can modulate the epithelial to mesenchymal transition, contributing to the control of the progression of mammary tumor cells. These findings underscore the potential of compound **3** as an effective antitumor agent against BC, highlighting the broader antitumor activity of the phenylsulfonylpiperazine class.

## 4. Materials and Methods

### 4.1. Chemistry

The preparation, purification and characterization of compounds **1**–**20** were performed according to previously reported methods [19,20].

### 4.2. Cell Culture

Four different breast cells lines were used in this study: MCF-10A (non-tumorigenic), MCF7 (ER+ BC), MDA-MB453 (HER2+ BC) and MDA-MB231 (TNBC). All lineages were obtained from the American Type Culture Collection (ATCC; Manassas, VA, USA) and maintained at a temperature of 37 °C according to the supplier’s recommendations. Only MDA-MB231 cells were not kept in an atmosphere of 5% CO_2_. MCF-10A was grown in Dulbecco’s Modified Eagle’s Medium (DMEM F12, Gibco, ThermoFisher Scientific, Waltham, MA, USA) supplemented with 20 ng/mL epidermal growth factor (Gibco, ThermoFisher Scientific, Waltham, MA, USA), 0.5 μg/mL hydrocortisone (Sigma-Aldrich, Sigma-Aldrich, St. Louis, MO, USA) and 10 μg/mL insulin (Gibco, ThermoFisher Scientific, Waltham, MA, USA). MCF7 was cultivated in Roswell Park Memorial Institute Medium (RPMI-1640, Gibco, ThermoFisher Scientific, Waltham, MA, USA), MDA-MB453 in IMDM and MDA-MB231 in Leibovitz’s medium (L-15, Gibco, ThermoFisher Scientific, Waltham, MA, USA). All media were supplemented with 10% fetal bovine serum (FBS, Gibco, ThermoFisher Scientific, Waltham, MA, USA) and 50 µg/mL of gentamicin (Cultilab, Campinas, SP, Brazil). The cells were confirmed to be free of mycoplasma and passaged using a 0.25% trypsin-EDTA solution when confluency reached 80–90%.

### 4.3. Cell Viability

Cell viability was determined by spectrophotometric quantification of MTT (3-[4,5-dimethyl-thiazol-2-yl]-2,5-diphenyltetrazolium bromide—Invitrogen. Waltham, MA, USA) reduction to formazan crystals. Cells were seeded at the density of 1 × 10^4^ cells/well (MCF-10A, MDA-MB453), 1.5 × 10^4^ cells/well (MDA-MB231) and 3 × 10^3^ cells/well (MCF7) in 96-well culture dishes and incubated with different concentrations of the compounds (1.25, 2.5, 5, 10, 20, 40, 80 and 160 µM) for 48 h. The medium was than replaced by MTT solution (0.5 mg/mL) and plates were kept at 37 °C for 4 h. After the aspiration of the media, the formazan crystals were diluted with 100 μL of dimethyl-sulfoxide (DMSO)/well and the absorbance at 570 nm was measured using a spectrophotometer (Thermo Plate TP-Reader, Waltham, MA, USA). Cytotoxicity was calculated using the following formula: Cytotoxicity (%) = [absorbance of cells treated with the compounds/absorbance of cells treated with DMSO) × 100]. The IC_50_ was determined for each compound and was defined as the concentration required to inhibit 50% of cell growth, compared to the control sample (cells treated with DMSO 0.05%).

### 4.4. ABTS Radical Scavenging Assay

The ABTS^+^ radical cation scavenging efficacy for compound **3** was determined according to the method described earlier [49] with some modifications. Briefly, an equal amount of 7 mM ABTS^+^ (2,20-azino-bis(3-ethylbenzothiazoline-6-sulfonic acid)) stock solution was mixed and kept in the dark at room temperature for 12 h with a 140 mM potassium persulfate stock solution. The ABTS^+^ was then diluted in ethanol until it reached a UV absorption value of 0.700 (±0.200) at 734 nm. Compound **3** (10, 20, 40, 80 and 160 µM) was mixed with ABTS^+^ in 96-well plates, and absorbance was read at 415 nm using a microplate reader (Thermo Plate TP-Reader, ThermoFisher Scientific, Waltham, MA, USA). The scavenging capability of ABTS^+^ radical was expressed as Trolox (0.1 to 2 mM) equivalents.

### 4.5. Colony Formation

MCF7 cells were seeded at a density of 500 cells per well in 6-well culture. The following day, the cells were treated with compound **3** (1.125, 2.25 and 4.5 µM) for 24 and 48 h. The medium was removed and, after 12 days, cells were washed 3 times with 1 × Phosphate-buffered saline (PBS), fixed with formaldehyde (4% *v*/*v*) and stained with crystal violet solution (0.5% *v*/*v*). Plates were scanned using L-Pix (Loccus Biotecnologia, Cotia, SP, Brazil) to obtain the images, incubated with 300 µL of acetic acid 33% *v*/*v*, and 100 µL of this solution was transferred to 96-well plates to evaluate the absorbance at 570 nm in a microplate reader (Thermo Plate TP-Reader, ThermoFisher). The number of colonies per well was calculated in relation to the untreated cells (considered as 100% of colony formation). 

### 4.6. Migration Assay

Wound-healing analysis was applied to test cell migration ability. MCF7 cells were seeded in a 6-well plate at a density of 1 × 10^6^ cells/well and, after confluence, a “wound” in the monolayer was introduced by a sterilized 200 μL pipet tip. Cells were washed with PBS and treated with compound **3** (1.125, 2.25 and 4.5 µM) for 24 or 48 h in a serum-free medium. Cells were photographed at 0 h, 24 or 48 h under a light microscope (Evos^®^, ThermoFisher Scientific, Waltham, MA, USA) at 10× objective using the same randomly chosen fields. The cell-free area was measured by ImageJ v. 1.54 g [50] software and migration in the control well was considered to be 100%.

### 4.7. qPCR Analysis

MCF7 cells were cultivated in 6-well plates and treated with 4.5 µM of **3** for 48 h. Total RNA was extracted using Trizol reagent (Invitrogen), according to the manufacturer’s protocol. The quality of extracted RNA was verified by the spectrophotometric readings at 260 and 280 nm (Nanodrop 1000-ThermoFisher), and the first strand of cDNA was synthesized from 1 µg of total RNA via reverse transcription using GoScript™ Reverse Transcription Mix (Promega, Madison, WI, EUA). qPCR analysis was performed using 5.0 μL of Power SYBR Green PCR Master Mix (Applied Biosystems, Carlsbad, CA, USA) and run on a StepOnePlus Systems (Applied Biosystems, Carlsbad, CA, USA) device with the following cycling parameters: 40 cycles of 95 °C for 10 s and 60 °C for 30 s. The comparative Cq was used to find the relative expression of *CDH1* (E-caderina), *ENTPD1* (ectonucleoside triphosphate diphosphohydrolase 1), *ENTPD2* (ectonucleoside triphosphate diphosphohydrolase 2), *ENTPD3* (ectonucleoside triphosphate diphosphohydrolase 3), *ENTPD5* (ectonucleoside triphosphate diphosphohydrolase 1) and *KRT10* (keratin 10) genes, after relative standard curve optimization. β-2-microglobulin (*β2M*) was used as a reference gene. The primer sequences are described in Table 3.

### 4.8. Statistical Analysis

Data were presented as the mean ± standard deviation (SD) and analyzed using GraphPad Prism 9.0 software (GraphPad Software Inc., La Jolla, CA, USA). All data were assayed in triplicate. Statistical analysis was performed using one-way ANOVA, Tukey´s HSD post hoc test and Student’s *t*-test. The IC_50_ was calculated using non-linear regression and the selectivity index (SI) was calculated by the ratio between the IC_50_ values corresponding to the non-tumor cell lineage (MCF-10A) and the IC_50_ values corresponding to the BC cell lines. Statistical significance was accepted at *p* values < 0.05.

## 5. Conclusions

Our results highlight the importance of the phenylsulfonylpiperazines class of compounds in the search for effective antitumor agents. The synthesis and biological evaluation of a library containing 20 compounds revealed that compound **3** was the most active and selective compound to luminal BC cells, standing out as a promising candidate for future assays. In addition to its relevant cytotoxicity to MCF7 lineage, compound **3** demonstrated antiproliferative and antimigratory activity by increasing *CDH1* expression. These findings suggest that compound **3** can help in the treatment of luminal tumors, requiring additional studies to uncover the additional mechanism of action of this compound and to evaluate its pharmacokinetic profile. Additionally, these results indicate that new derivatives of this chemical class, incorporating the tetrazole fragment or other aromatic azole nuclei, should be designed, synthesized and evaluated against BC. This approach could lead to the discovery of even more promising compounds.

## Figures and Tables

**Table 1:** Half-maximal inhibitory concentration (IC_50_) and selectivity index (SI) values for the treatments with their indicated compounds. Mammary cell lines were treated with increasing concentrations (1.25–160 μM) of each compound for 48 h. Cell viability was determined using the MTT assay. SI values were calculated by dividing the IC_50_ value for non-tumor cell line MCF10A by the IC_50_ values for tumor cells (MCF7, MDA-MB-231 and MDA-MB-453).

Compound	Structure	IC_50_ (µM)				SI		
		MCF-10A	MCF7	MDA-MB-231	MDA-MB-453	MCF7	MDA-MB-231	MDA-MB-453
**1**	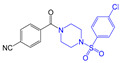	>160	>160	84.90	>160	-	>1.88	-
**2**	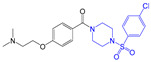	88.20	>160	61.34	50.51	NS	1.44	1.75
**3**	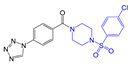	>160	4.48	83.02	80.00	>35.6	>1.93	>2.00
**4**	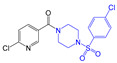	>160	>160	112.50	>160	-	>1.42	-
**5**	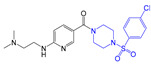	>160	82.89	>160	>160	>1.93	-	-
**6**	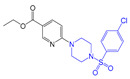	>160	>160	53.10	>160	-	>3.01	-
**7**	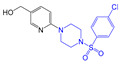	91.17	>160	107.30	>160	NS	0.85	NS
**8**	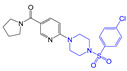	86.01	153.90	134.80	92.52	NS	NS	NS
**9**	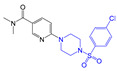	63.89	>160	>160	>160	NS	NS	NS
**10**	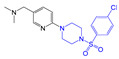	108.80	62.03	>160	48.38	1.75	NS	2.25
**11**	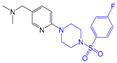	>160	20.00	>160	108.60	>8.00	-	>1.47
**12**	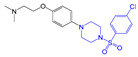	68.34	49.89	>160	136.10	1.37	NS	NS

NS: the compound was more active in the non-tumor cell line. -: the compound was not cytotoxic at the concentrations tested and it was not possible to calculate the SI.

**Table 2:** Fold change in expression levels of mRNAs of different biomarkers in MCF7 (luminal breast cancer). Treatments were compared to untreated cells (control). *p* value represented by *** (*p* < 0.001) using Student’s *t*-test.

Gene	Compound 3 (µM)	Fold Change
*CDH1*	1.125	1.874
	2.25	1.045
	4.5	4.034 ***
*ENTPD1*	1.125	1.102
	2.25	1.331
	4.5	1.006
*ENTPD2*	1.125	1.374
	2.25	1.417
	4.5	1.109
*ENTPD3*	1.125	1.641
	2.25	1.074
	4.5	1.229
*ENTPD5*	1.125	2.046
	2.25	1.437
	4.5	1.306
*KRT10*	1.125	1.309
	2.25	1.913
	4.5	1.383
Color key:	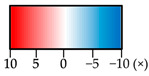	

**Table 3:** Primers used in qPCR analysis.

Gene	Forward Primer (5′-3′)	Reverse Primer (5′-3′)	Amplicon (bp)
*β2M*	CCTGCCGTGTGAACCATGT	GCGGCATCTTCAAACCTCC	94
*CDH1*	CTGGCGTCTGTAGGAAGGC	GCTGGCTCAAGTCAAAGTCCTG	240
*ENTPD1*	TGTGGTGGAGAGGAGCCTCA	GCTGAACCACCTTGTTTTCTGAC	142
*ENTPD2*	TGCTGGAGAACTTCATCAAGTACG	CAAAAGTGATCTGGGTAGAGGCAC	108
*ENTPD3*	CTCCCTCCAGGACTGAAGTATGG	GCATACACTCCTCAAAGGCTCTG	202
*ENTPD5*	CAAGGCTCTGCTCTTTGAGGTAA	CGTGATTTGGGTGGAGGCT	202
*KRT10*	TAGGGTGCTGGATGAGCTGAC	TCGAAGGTCTTTCATTTCCTCC	118

## Data Availability

The data that support the findings of this study are available from the corresponding author upon reasonable request.

## Supplementary Materials

The following supporting information can be downloaded at https://www.mdpi.com/article/10.3390/molecules29184471/s1. Table S1: Compounds with half-maximal inhibitory concentration (IC_50_) > 160 µM. Cells lines were treated for 48 h and viability was determined using the MTT assay. Figure S1: Graphical representation of compounds with half-maximal inhibitory concentration (IC50) < 160 µM in at least one of the tested cell lines, compounds **1**–**12** (A-L). Cell lines were treated for 48 h and viability was determined using the MTT assay. Figure S2: Graphical representation of compounds with half-maximal inhibitory concentration (IC50) > 160 µM, compounds **13**–**20** (A-H). Cell lines were treated for 48 h and viability was determined using the MTT assay. Figure S3: Graphical representation of the cytotoxic activity of compound **3** in the cell lines tested.
