# Supplementary material for: A Novel Compound from the Phenylsulfonylpiperazine Class: Evaluation of In Vitro Activity on Luminal Breast Cancer Cells
Source: Molecules. 2024 Sep 20;29(18):4471. doi: 10.3390/molecules29184471 (PMC11433764; doi:10.3390/molecules29184471)
Supplement: Supplementary file 1 [file molecules-29-04471-s001.zip › molecules-3138596-supplementary.pdf]

Supplementary Materials

A Novel Compound from the Phenylsulfonylpiperazine Class: Evaluation of In Vitro Activity on Luminal Breast Cancer Cells

Fernanda Cardoso da Silva [1], Ana Clara Cassiano Martinho [2], Helen Soares Valença Ferreira [1], Raoni Pais Siqueira [1], Vinicius Marques Arruda [1,3], Joyce Ferreira da Costa Guerra [3], Maria Laura dos Reis de Souza [2], Emanuelly Silva Landin [2], Celso de Oliveira Rezende Júnior [2] and Thaise Gonçalves de Araújo [1,4,*]

[1] Laboratory of Genetics and Biotechnology, Institute of Biotechnology, Universidade Federal de Uberlândia, Patos de Minas 38700-002, MG, Brazil; fernanda.cardoso95@yahoo.com (F.C.d.S.); helensvalenca@gmail.com (H.S.V.F.); raoni.siqueira@ufu.br (R.P.S.); viniciusmarquesarruda@gmail.com (V.M.A.)
[2] Laboratory of Drug Candidate Synthesis, Institute of Chemistry, Universidade Federal de Uberlândia, Uberlândia 38400-902, MG, Brazil; anaclaracm25@gmail.com (A.C.C.M.); marialaura1200@hotmail.com (M.L.d.R.d.S.); emanuelly.landin@ufu.br (E.S.L.); celso@ufu.br (C.d.O.R.J.)
[3] Laboratory of Biochemistry, Institute of Biotechnology, Universidade Federal de Uberlândia, Patos de Minas 38700-002, MG, Brazil; joyceguerra@ufu.br (J.F.d.C.G.)
[4] Laboratory of Nanobiotechnology Prof. Dr. Luiz Ricardo Goulart Filho, Institute of Biotechnology, Universidade Federal de Uberlândia, Uberlândia 38405-302, MG, Brazil
* Correspondence: tgaraujo@ufu.br; Tel.: +55-34-38142027

Table S1: Compounds with half-maximal inhibitory concentration (IC_{50}) > 160 μM. Cells lines were treated for 48 h and viability was determined using the MTT assay.

Compound	Structure	IC50 (mM)			
		MCF-10A	MCF7	MD-AMB 231	MD-AMB 453
13	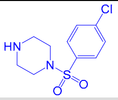	> 160	> 160	> 160	> 160
14	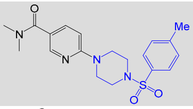	> 160	> 160	> 160	> 160
15	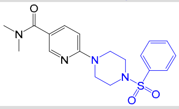	> 160	> 160	> 160	> 160
16	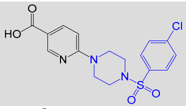	> 160	> 160	> 160	> 160
17	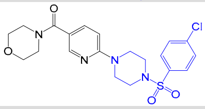	> 160	> 160	> 160	> 160
18	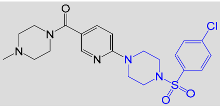	> 160	> 160	> 160	> 160
19	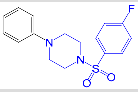	> 160	> 160	> 160	> 160
20	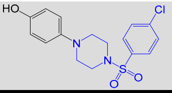	> 160	> 160	> 160	> 160

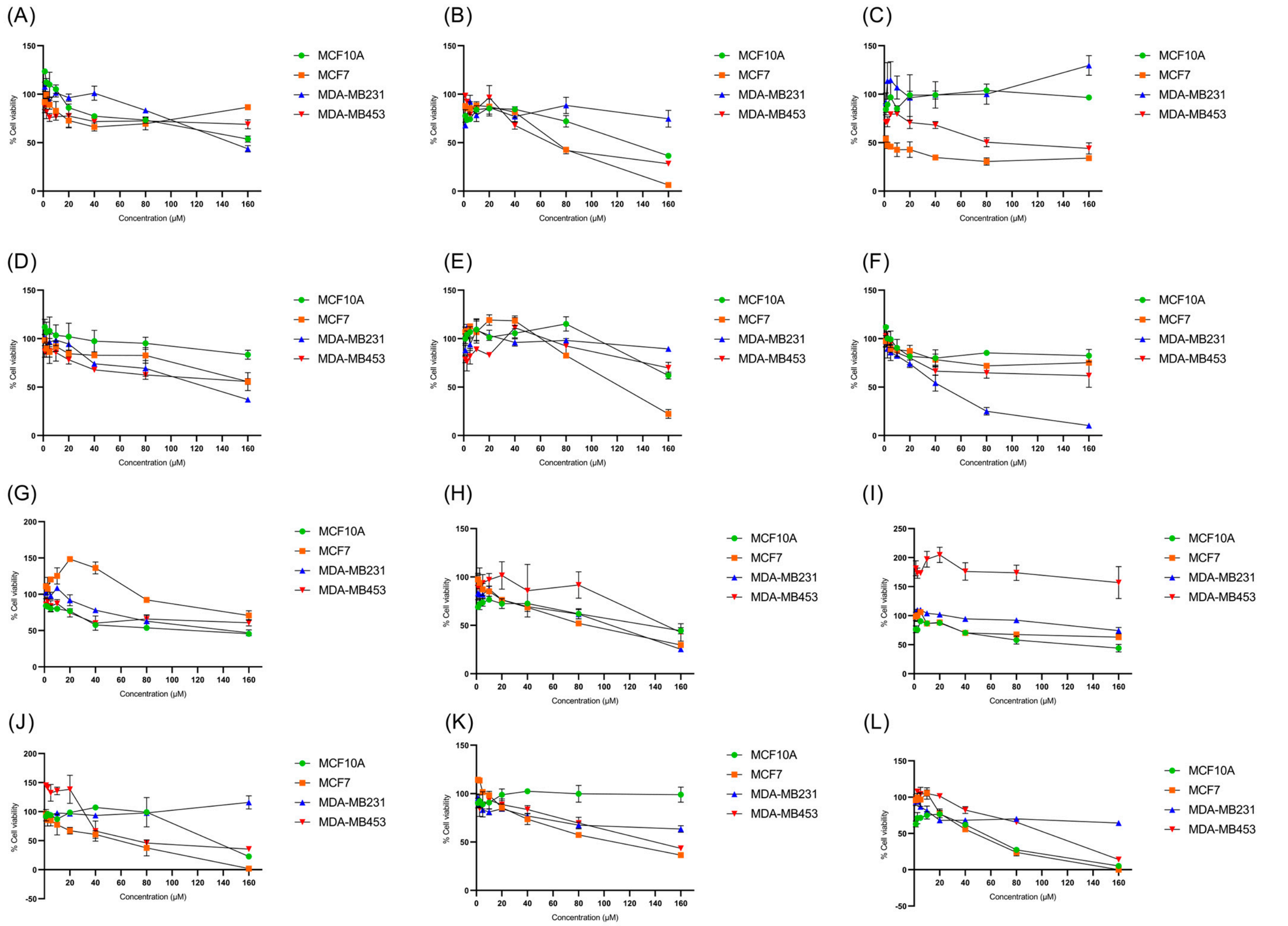

Figure S1: Graphical representation of compounds with half-maximal inhibitory concentration (IC50) < 160 μM in at least one of the tested cell lines, compounds **1 - 12** (A-L). Cell lines were treated for 48 h and viability was determined using the MTT assay.

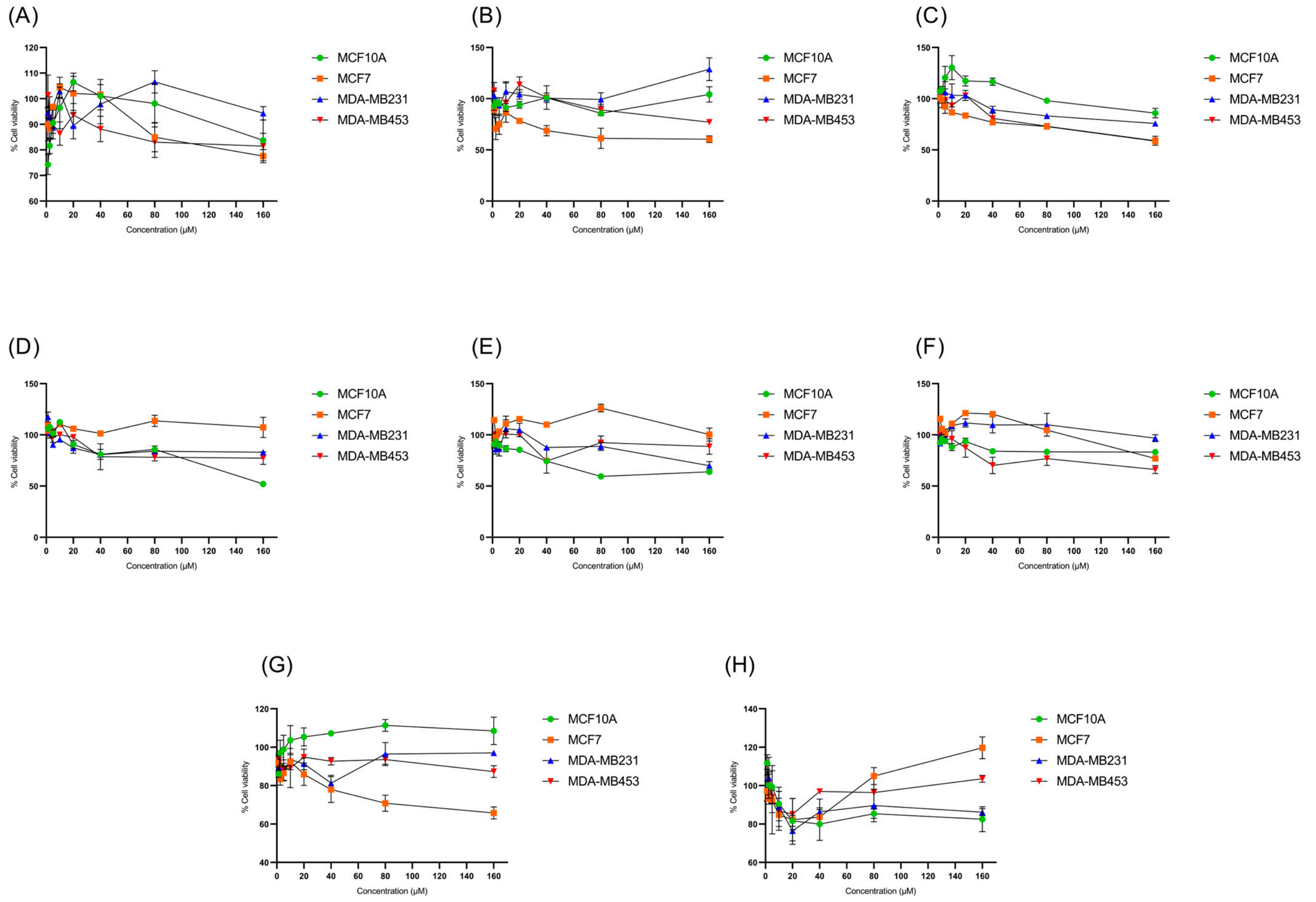

Figure S2. Graphical representation of compounds with half-maximal inhibitory concentration (IC50) > 160 μM, compounds **13 - 20** (A-H). Cell lines were treated for 48 h and viability was determined using the MTT assay.

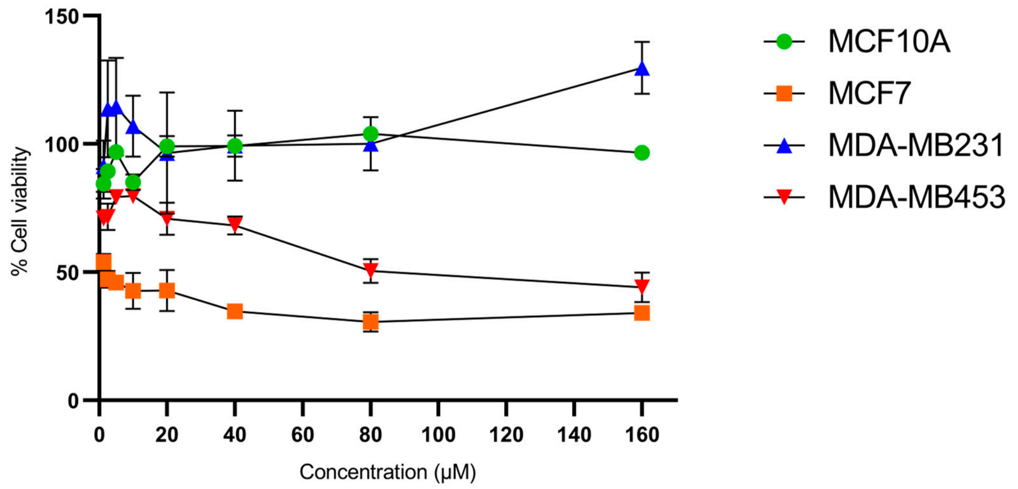

Figure S3: Graphical representation of the cytotoxic activity of compound **3** in the cell lines tested.
